# Sub-10 μm-Thick Ge Thin Film Fabrication from Bulk-Ge Substrates via a Wet Etching Method

**DOI:** 10.1021/acsomega.3c07490

**Published:** 2023-12-12

**Authors:** Liming Wang, Ying Zhu, Rui-Tao Wen, Guangrui Xia

**Affiliations:** † Department of Materials Engineering, The University of British Columbia, Vancouver, BC V6T 1Z4, Canada; ‡ Department of Materials Science and Engineering, Southern University of Science and Technology, Shenzhen 518055, China

## Abstract

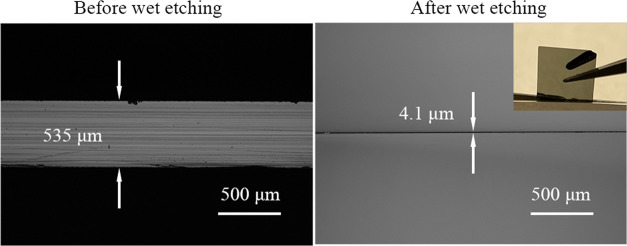

Low-defect density Ge thin films are crucial for studying the impact of defect density on the performance limits of Ge-based optical devices (optical detectors, LEDs, and lasers). Ge thinning is also important for Ge-based multijunction solar cells. In this work, Ge wet etching using three acidic H_2_O_2_ solutions (HF, HCl, and H_2_SO_4_) was studied in terms of etching rate, surface morphology, and surface roughness. HCl–H_2_O_2_–H_2_O (1:1:5) was demonstrated to wet-etch 535 μm-thick bulk-Ge substrates to 4.1 μm with a corresponding RMS surface roughness of 10 nm, which was the thinnest Ge film from bulk-Ge via a wet etching method to the best of our knowledge. The good quality of pre-etched bulk-Ge was preserved, and the low threading dislocation density of 6000–7000 cm^–2^ was maintained after the etching process. This approach provides an inexpensive and convenient way for accurate Ge substrate thinning in applications such as multijunction solar cells and sub-10 μm-thick Ge thin film preparation, which enables future studies of low-defect density Ge-based devices such as photodetectors, LEDs, and lasers.

## 1 Introduction

As Si-based transistors scale down and become faster with every successive generation, the low speed and high energy consumption problems of conventional metal interconnects in Si ICs are becoming the performance bottleneck.^1−3^ Si-compatible optical interconnects are in great need to address this issue. Up to date, most of the Si-compatible optical components required for on-chip optical interconnects, such as modulators,^4^ photodetectors,^5^ and waveguides,^6^ are already available. The last missing piece is a light source, especially a laser. Although InAs/GaAs quantum dot lasers monolithically grown on Si have been realized, due to material contamination issues, it may take a long time and high cost for those III–V materials to enter mainstream Si-processing facilities.

Ge is an indirect-band gap semiconductor, which is inferior in light emission. However, it is the most Si-compatible semiconductor. Ge is already used in mainstream Si fabrication facilities and plays an important role in Si photonics, such as detectors and modulators. With n-type doping and strain engineering, edge-emitting Ge lasers were first demonstrated in 2010 and have been investigated by a few academic groups.^7−9^ However, early Ge laser’s performance was far below suitable commercial standards.

Theoretical studies on epitaxial Ge (epi-Ge)-on-Si lasers indicated that with design optimization, threshold current densities and wall-plug efficiencies of epi-Ge lasers could be greatly improved.^10,11^ A 3 dB bandwidth of 33.94 GHz at a biasing current of 270.5 mA was predicted after Ge laser structure optimization with a defect-limited carrier lifetime of 1 ns.^12^ So far, the main obstacle to achieving this potential lies in the poor epitaxial Ge quality on Si substrates due to the lattice mismatch between Ge and Si. The thread dislocation density (TDD) in Ge grown on Si is typically in the range of 10^6^–10^9^ cm^–2^. High TDD results in a short minority carrier lifetime, high lasing threshold, poor reliability, and low efficiency, which greatly limit the performance of lasers fabricated on epi-Ge wafers. In comparison, bulk-Ge crystals, such as Ge wafers, have the highest material quality, and the TDD for bulk-Ge wafers is commonly below 10^4^ cm^–2^.

What is the ultimate performance potential of Ge lasers? Are Ge lasers a feasible technology solution to the long-existing Si-compatible laser problem? Our long-term objective is to answer these questions experimentally using the highest-quality Ge, bulk-Ge, which has not been used for Ge laser preparation before. To make a Ge laser from bulk-Ge, the first step is to obtain Ge thin films of micron scale from bulk-Ge wafers of a few hundred μm thickness, which is the goal of this work. Besides Ge lasers, Ge thin films are also used in photodetectors and LEDs.^13−17^ Ge thin films with thicknesses of several μm prepared from bulk-Ge wafers are of great interest for solar cell application but have not yet been accomplished to the best of our knowledge.^18−20^ Meanwhile, Ge thin films have been demonstrated to be a good structure for introducing large mechanical tensile strain to achieve a direct band to band emission.^21−23^ Bulk-Ge-based thin films are also helpful to study how the defect density in Ge can impact the performances of these devices as well.

While a smart-cut method was proposed to obtain a Ge thin film on the Si substrate,^24,25^ solution-based methods such as wet etching to thin Ge are much cheaper and more accessible to get Ge thin films, especially in the early R&D stage. With a high-quality bulk-Ge crystal, it is possible to get a high-quality Ge thin film for potential optoelectronic applications discussed above.

As the pioneering transistor material, Ge’s first wet etching study dates back to 1955, when Paul. R. Camp studied the etching rates of Ge with solutions composed of H_2_O_2_, HF, and water as a function of etchant composition, crystal orientation, and impurity.^26^ More etchants for Ge wet etching were studied in the following years, and the related etching rates of Ge for different solutions are well summarized in the literature.^27,28^ However, there are only three reports on the preparation of Ge thin films from bulk-Ge via wet etching, which are summarized in Table 1.

**Table 1:** Existing Studies for Ge Thin Films from Bulk-Ge via Wet Etching

	**etchant**	**target thickness (**μm**)**	**thickness obtained (**μm**)**	**surface roughness**	**application**	**refs**
**1**	HF:H_2_O_2_:H_2_O = 1:1:1		28		Ge band gap study	(23)
**2**	H_3_PO_4_:H_2_O_2_:H_2_O = 1:6:3	5–10	85		solar cell	(29)
**3**	H_3_PO_4_:HNO_3_:HF	5–10	80	0.42 nm	solar cell	(18)

The goal of this work is to develop wet etching recipes that can obtain low-defect Ge thin films from bulk-Ge wafers within 7 days of etching. The desired Ge thin films should have the following:

### 1.1 Thickness Less than 10 μm

It was reported that the direct-gap photoluminescence (PL) is difficult to be observed in thick bulk-Ge samples due to the reabsorption of the emitted photons.^30^ Decreasing the thickness to less than 10 μm is needed to decrease the reabsorption from Ge.^31^

### 1.2 Surface Roughness Less than 10 nm

The common surface roughness of epitaxial Ge thin films is in the nm scale. The choice of 10 nm as the upper limit is to match the roughness of epitaxial Ge films. Rough surfaces increase surface recombination and lower minority carrier lifetimes, which are not desired.

### 1.3 Threading Dislocation Density (TDD) Less than 10^4^ cm^–2^

The threading dislocation of bulk-Ge is commonly less than 10^4^ cm^–2^, which should be preserved after the wet etching.

## 2 Experiments

The beginning substrates were 4-inch n-type (0.173–0.25 Ohms*cm at 295 K) double side polished (100) Ge Czochralski wafers that were obtained commercially. The surface roughness of the pre-etched Ge wafer is 1.6 nm. The wafers were diced into 1 cm × 1 cm pieces before the etching process. All the Ge pieces were cleaned sequentially with acetone, isopropyl alcohol, and deionized (DI) water and dried with N_2_ gas. All the wet etching was done in a wet bench with good ventilation in a class 10,000 cleanroom with the temperature controlled at 21 °C.

### 2.1 Etchant and Etch Recipe Selection

As the initial thickness of the Ge wafer is 535 μm, the minimum etching rate should be 50 nm/min to obtain a sub-10 μm Ge thin film in 1 week under a uniform etching rate. Based on the etch rate reported from the literature,^25^ the only suitable choices were NH_4_OH-based H_2_O_2_ solution, HCl-based H_2_O_2_ solution, and H_2_SO_4_-based H_2_O_2_ solution. Acidic H_2_O_2_ solutions have been studied and widely used for Ge etching.^32^ Hence, H_2_SO_4_- and HCl-based H_2_O_2_ solutions were selected. In cleanrooms, the nanostrip solution (Nanostrip 2X: 85% H_2_SO_4_, ≤ 1% H_2_O_2_, manufactured by KMG) is more frequently used than the concentrated H_2_SO_4_ solution (96%). Therefore, we used nanostrip solutions instead of concentrated H_2_SO_4_. One more solution selected was an HF-based H_2_O_2_ solution, which was used to obtain a 28 μm-thick Ge.^26^ In total, three types of solutions were chosen for Ge wet etching:

(1) HCl-based H_2_O_2_ solutions consisting of HCl solution (37%, manufactured by J.T.Baker), H_2_O_2_ solution (30%, manufactured by J.T.Baker), and DI water.
(2) Nanostrip-based H_2_O_2_ solutions consisting of Nanostrip 2X solution (85% H_2_SO_4_, ≤1% H_2_O_2_, KMG), H_2_O_2_ solution (30% by J.T.Baker), and DI water.
(3) HF-based H_2_O_2_ solutions consisting of HF solution (49%, manufactured by J.T.Baker), H_2_O_2_ solution (30%, J.T.Baker), and DI water.

To simplify the description of the etch solutions, we use the acid name plus a volume ratio (X: Y: Z) to denote an etch solution made of X parts of the acid product specified, Y parts of H_2_O_2_, and Z parts of DI water. For example, an HCl (X:Y:Z) solution means a solution consisting of X parts of HCl solution (37%, J.T. Baker), Y parts of H_2_O_2_ (30%), and Z parts of water.

### 2.2 Etch Recipe Optimization for Better Surface Morphology

To produce a Ge thin film with a thickness of ≤10 μm, the surface morphology is a crucial factor. A thin film with high roughness before and during wet etching is more prone to breaking into pieces before reaching the desired thickness due to the preferential etching near defects such as surface scratches and dislocations. To check how the volume ratio X:Y:Z influences the postetching morphology, all three types of solutions were prepared with ratios of 1:0:0, 1:1:1, 1:1:5, 1:1:10, and 1:1:20. To conduct the wet etching, each Ge piece (1 cm × 1 cm) was placed on the bottom of a beaker and the etchant solution with a volume of ∼35 mL was added into the beaker for a certain etching time (25 min for HF-based solutions due to the fast etch rates, 24 h for HCl and nanostrip-based solutions). This sample placement method resulted in single-sided etching. The thickness before and after the etching process was checked with a Beslands micrometer.

Because surface morphology plays an important role in optoelectronic devices, in this work, we inspected the postetching Ge morphologies with a Nikon ECLIPSE LV150 optical microscope. This was chosen instead of an electron microscope because a larger imaging area of 3.5 mm^2^ was preferred to represent the overall morphology.

The 3D optical images were taken with an optical interferometer (Filmetrics Profilm3D optical surface profiler) to evaluate the surface roughness after etching. The optical interferometer is a good metrology tool for quantitatively measuring roughness for a large area (≥400 μm × 300 μm). If the measurement area is limited to the μm or nm scale, the results can be misleading. For example, one sample can be smooth in the μm or nm scale but rough in the sub-mm scale. The roughness in the sub-mm scale can result in sample etching or fracture. The volume ratio generating the lowest surface roughness for each solution was selected for Ge thin film preparation and characterizations, which was HCl (1:1:5) and nanostrip (1:1:10) (details in 3.2 and 3.3). HF solutions were eliminated due to high surface roughness, as discussed in 3.1.

### 2.3 Thin Film Preparation and Characterizations

In this step, Ge wafers were thinned to ≤10 μm using the two selected recipes: HCl (1:1:5) and nanostrip (1:1:10) and double-sided etching. Ge was placed vertically in the beaker on a small Teflon stand with double sides being etched at the same time to shorten the required etching time. The remaining thickness of the Ge thin film was checked with optical microscopy for the cross-section.

The surface roughness was evaluated with an optical interferometer, and the etching pit density (EPD) measurements were performed to obtain the TDD before and after etching using an etching solution. The EPD etchant which was a mixture of 100 mL of CH_3_COOH (≥99%, J.T.Baker), 40 mL of HNO_3_ (70%, J.T.Baker), 10 mL of HF (49%, J.T.Baker), and 30 mg of I_2_ (≥99.99%, Sigma-Aldrich) was selected according to the literature.^33^ An optical microscope was used to observe, count the etch pits, and calculate the etch pit density (EPD) with more than three positions being checked. The crystal quality before and after the etching was checked with high-resolution XRD (Bruker D8). The reflectance before and after the etching was measured with a film thickness measurement instrument F20 model by Filmetrics.

## 3 Results and Discussion

### 3.1 Optimization and Elimination of HF Solutions

**Figure 1:**
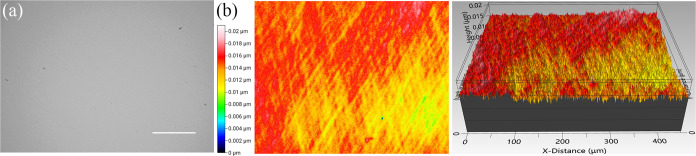
Optical images of (a) unetched virgin Ge wafer surface and 3D optical images of (b) unetched sample with Sq = 1.6 nm.

The optical image before the wet etching process is shown in Figure 1a, and the related surface roughness measured (Figure 1b) indicated that the unetched Ge had a roughness of approximately 1.6 nm with some minor polishing traces on the top. Owing to the high etching rates for HF-based solutions, the initial etching time was controlled to be 25 min for the recipe optimization. The postetching results for different ratios are shown in Table 2. The HF-only solution could not thin Ge down but was able to clean the Ge surface to obtain a low surface roughness of 1 nm.^34^ The HF solution (1:1:10) generated the lowest surface roughness. However, when the etching time was extended from 25 min to 4 h for the HF-based solution (1:1:10), the surface roughness increased drastically, which could be seen in Figure S1 with obvious cracks on the surface. Therefore, HF-based solutions were eliminated in the further thin film preparation.

**Table 2:**
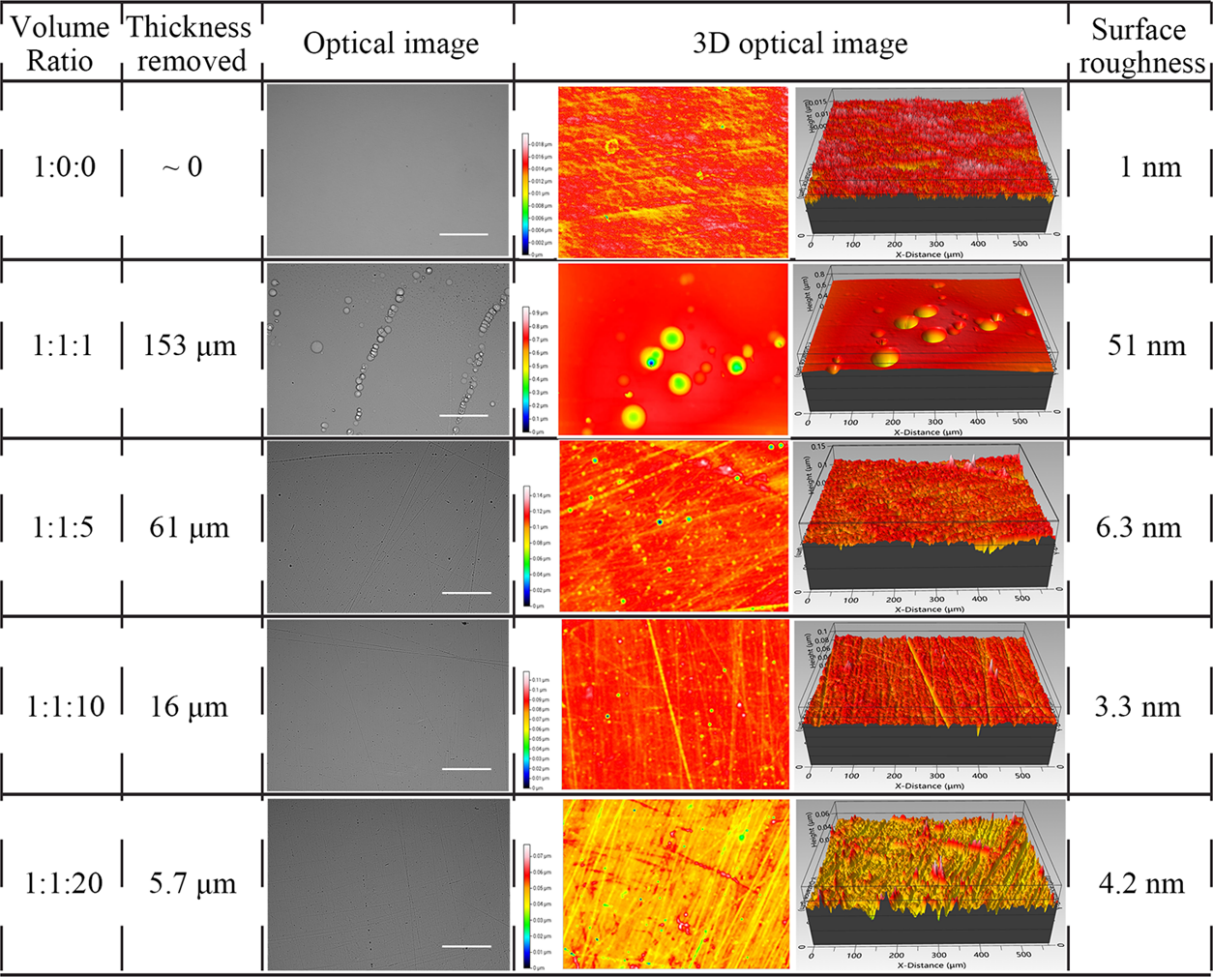
Optical Images, 3D Optical Images, Surface Roughness, and Thickness Removed under 25 min Etching from the HF-Based Solution with Different Ratios, Scale Bar = 500 μm

### 3.2 Optimization of HCl Solutions

The etching results for HCl solutions are shown in Table 3. After 24 h of etching with HCl (1:1:1), the thickness was reduced by 140 μm. Scratches and voids showed up with the surface roughness increased to 7.6 nm. As the ratio changed from 1:1:1 to 1:1:5, the surface roughness decreased to 6.3 nm and the etching rate reduced slightly to 130 μm/day. However, when the ratio increased to 1:1:10, the number of etching pits and the surface roughness increased sharply to 27.8 nm. The surface etched by HCl (1:1:20) became quite rough with a matte appearance under the optical microscope and was not able to be evaluated with the optical interferometer. Based on these observations, HCl (1:1:5) was selected for thin film preparation.

**Table 3:**
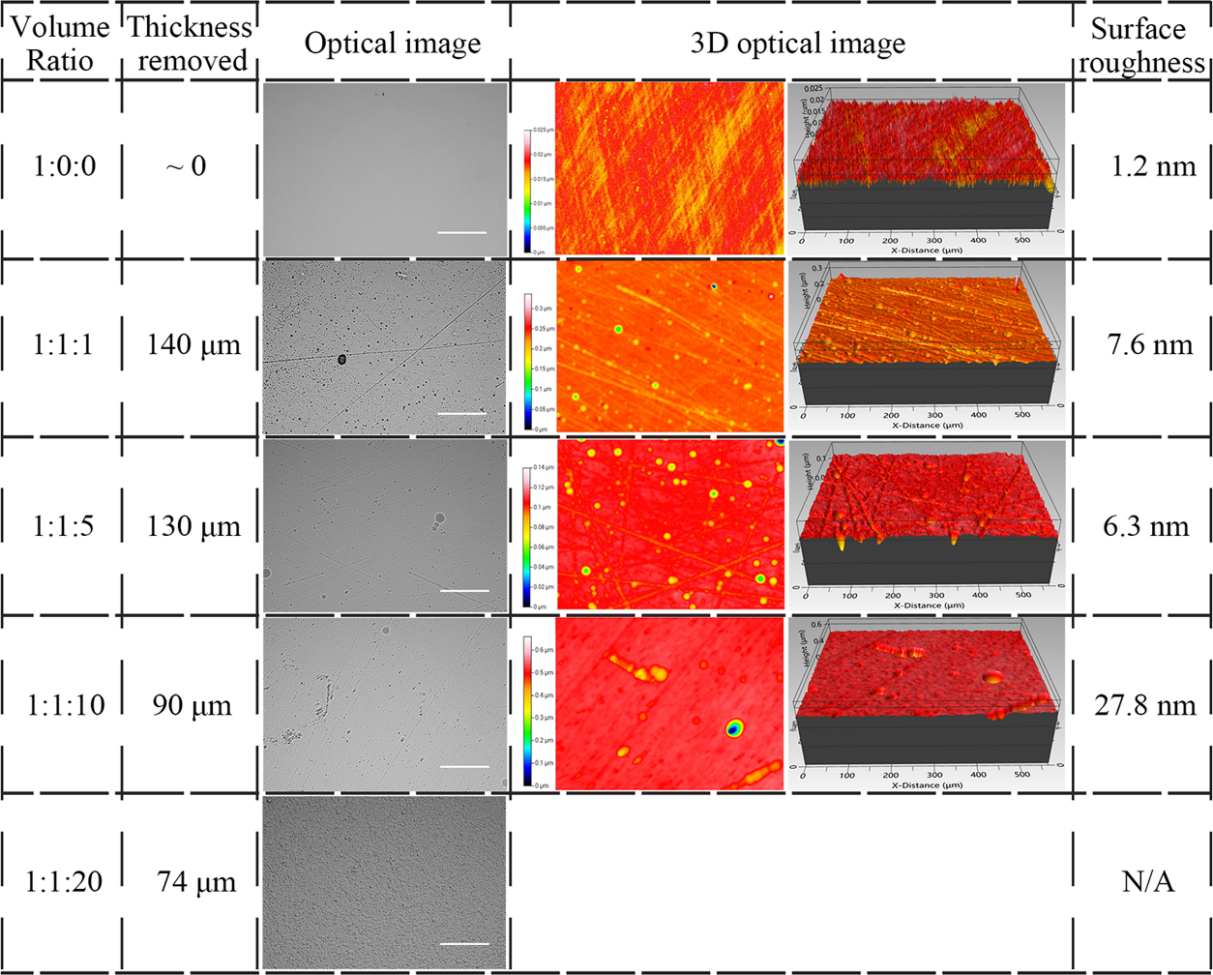
Optical Images, 3D Optical Images, Surface Roughness, and Thickness Removed after 24 h of Etching from the HCl-Based Solution with Different Ratios, Scale Bar = 500 μm

### 3.3 Optimization of Nanostrip Solutions

On the high H_2_SO_4_ limit, nanostrip (1:0:0) with no H_2_O_2_ or water, the etched Ge sample showed obvious scratches on the surface, with the surface roughness increased slightly to 2 nm. There was no obvious change of the thickness after 24 h of etching, indicating that Ge was roughened with little thickness loss. With the ratio change to 1:1:1, the etchant had a strong oxidative effect on the surface with oxidized particles on the surface. The surface was too rough to be measured using an optical interferometer. For the nanostrip (1:1:5) solution, obvious holes could be seen after etching, making the surface too rough to be measured with the optical interferometer in a vertical scanning interferometry mode. The etch recipe that generated the best surface quality was the nanostrip (1:1:10), and the etched surface is flat with minor voids on the surface with the lowest surface roughness of 3.8 nm. For the nanostrip (1:1:20), the surface roughness increased to 8 nm. According to these results, the nanostrip (1:1:10) was selected for thin film preparation (Table 4).

**Table 4:**
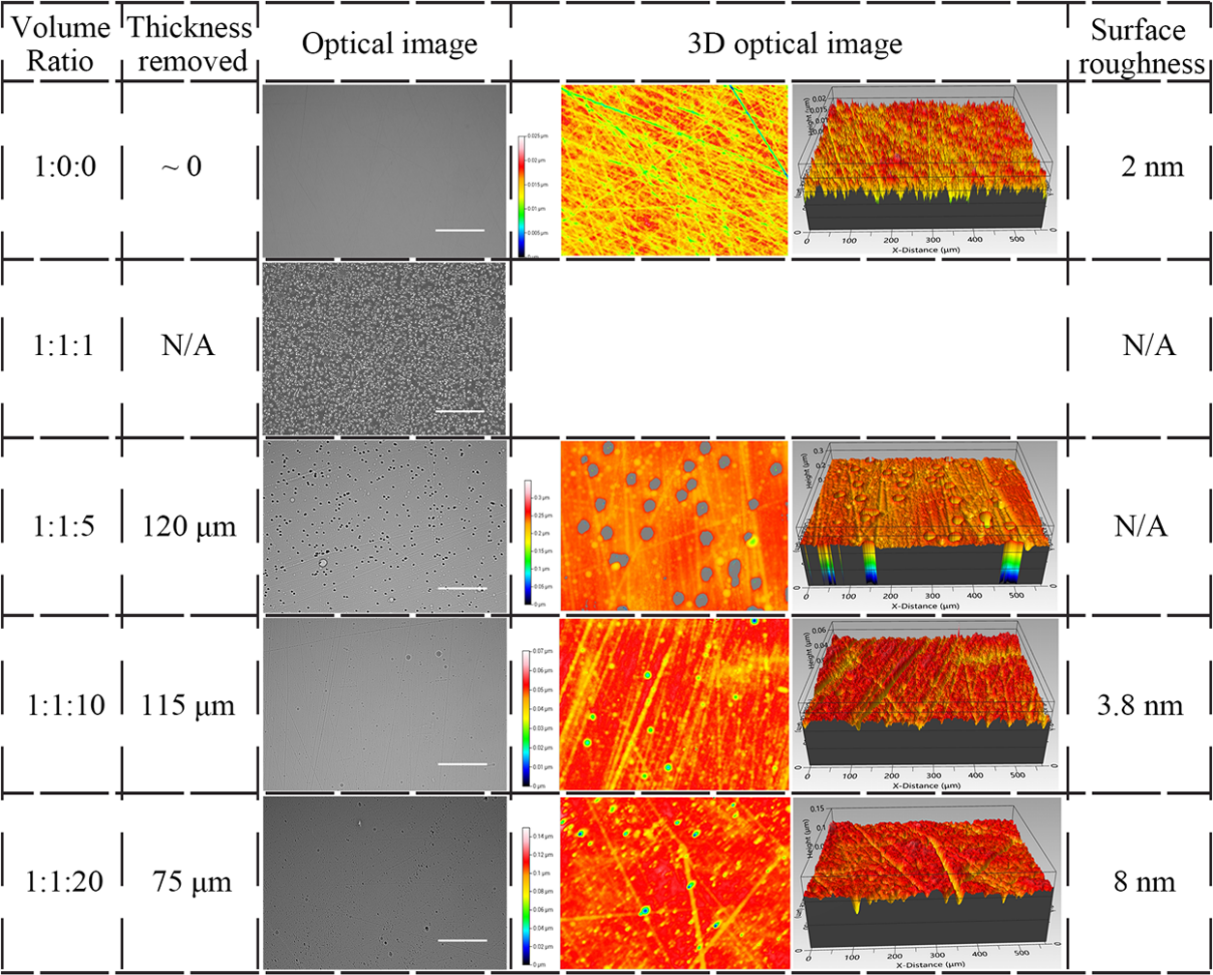
Optical Images, 3D Optical Images, Surface Roughness, and Thickness Removed under 24 h of Etching from the Nanostrip-Based Solution with Different Ratios, Scale Bar = 500 μm

### 3.4 Ge Thin Film Preparation by Double-Sided Etching with HCl (1:1:5) and the Nanostrip (1:1:10)

As discussed, HCl (1:1:5) and the nanostrip (1:1:10) were used to achieve the thinnest Ge films possible. To exclude the influence of the potential sediment during long-time etching and half the etching time, double-sided etching was used, where Ge was placed vertically in the beaker on a small Teflon stand (Figure S2). The surface morphology stayed almost the same for single-sided etching and double-sided etching (Figure S3), but the required etching time was shortened due to the etching on both sides. Ge film thicknesses were checked with optical microscopy.

**Figure 2:**
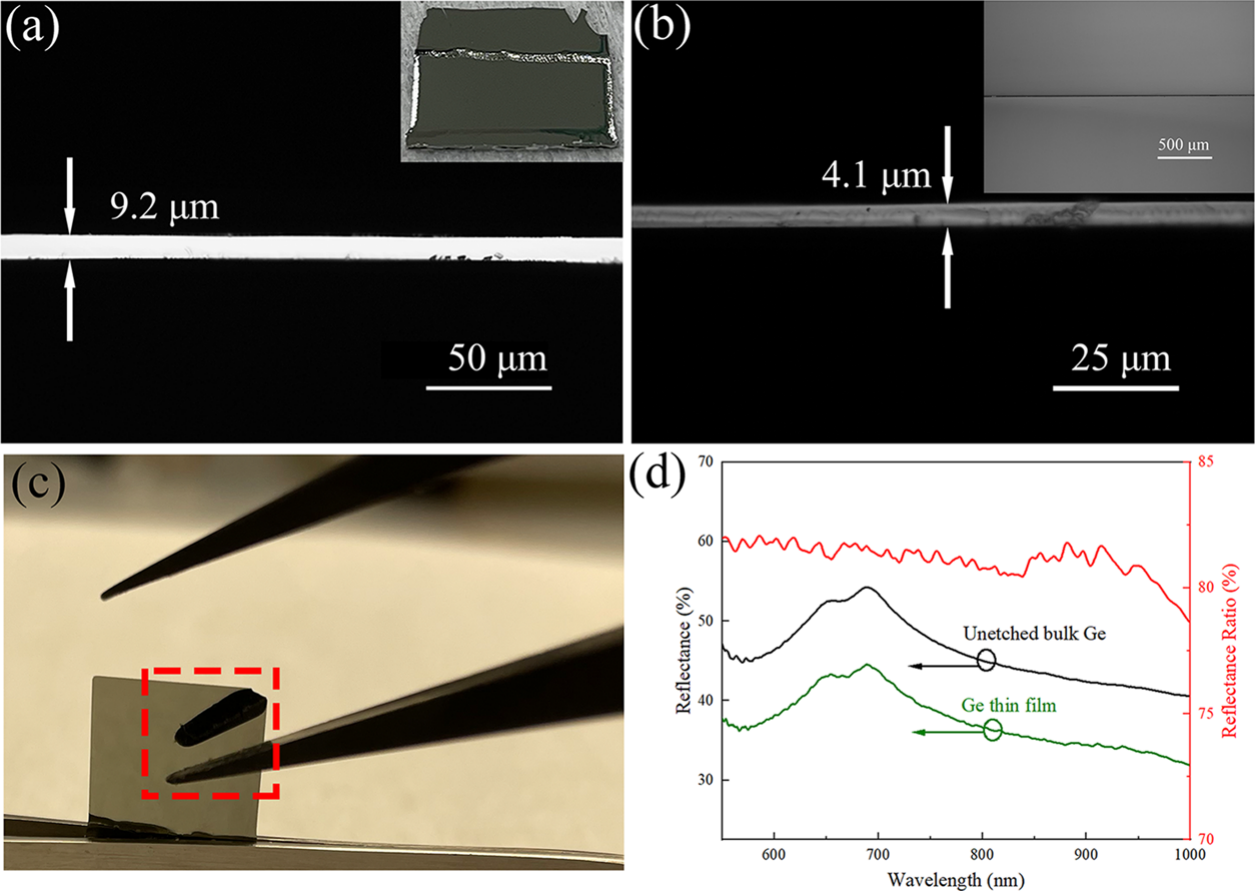
(a) Cross-section of the sample etched by the nanostrip (1:1:10) for 57 h and the photo (insert). (b) Cross-section of the sample etched by HCl (1:1:5) for 53 h and the top view (insert). (c) Picture of HCl (1:1:5)-etched Ge showing the reflection of a tweezer. (d) Reflectance curves and the reflectance ratio of unetched Ge vs HCl (1:1:5) 53 h-etched Ge thin film.

The final results of the thin films prepared are shown in Figure 2. Both the nanostrip (1:1:10) and HCl (1:1:5) were able to fabricate <10 μm Ge thin films. As shown in Figure 2a, double-sided etching by the nanostrip (1:1:10) for 57 h resulted in a thickness of 9.2 μm. The picture of the samples is shown on the top right. The thickness of Ge after HCl (1:1:5) 53 h etching was 4.1 μm (Figure 2b). A mirror-like surface was still kept for the HCl (1:1:5)-etched sample with the reflection of a tweezer seen (Figure 2c). The reflectance curves before and after the etching are shown in Figure 2d. More than 80% of the reflectance was preserved at the short wavelength side below ∼970 nm, with over 77% in the range between 970 and 1000 nm.

#### 3.4.1 Surface Roughness of the As-Etched Ge Thin Film

**Figure 3:**
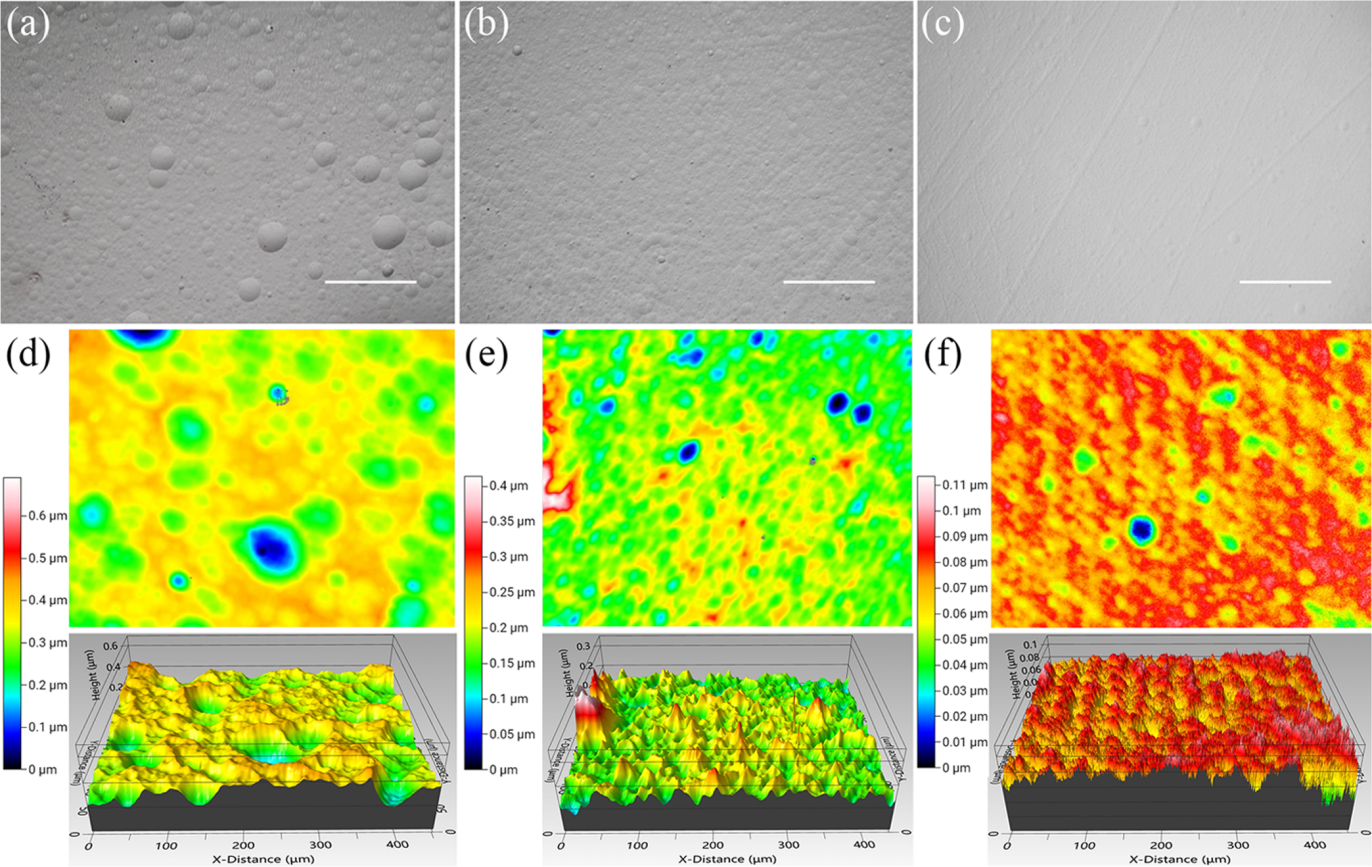
Optical images of (a) 57 h-nanostrip (1:1:10)-etched sample, (b) 51 h-nanostrip (1:1:10)-etched sample with agitation, and (c) 53 h-HCl (1:1:5)-etched sample without agitation. 3D optical images of (d) nanostrip (1:1:10)-etched sample, Sq = 60 nm, (e) nanostrip (1:1:10)-etched sample with agitation, Sq = 32 nm, and (f) HCl (1:1:5)-etched sample without agitation, Sq = 10 nm.

After nanostrip (1:1:10) double-sided etching for 57 h, the optical images showed a lot of hemispherical holes on the top (Figure 3a), and the surface roughness (Figure 3d) increased from 3.8 nm from the 24 h single-sided etching to 60 nm with surface holes of different sizes. This could be improved by an agitation (300 rpm) during the etching process where the surface etching hole sizes decreased (Figure 3b) and the surface roughness (Figure 3e) dropped to 32 nm. The HCl (1:1:5)-etched thin film had fewer etching holes and a flatter surface (Figure 3c), and the surface roughness (Figure 3f) was approximately 10 nm, much better than those etched by the nanostrip (1:1:10). This also explains the high reflectance of the HCl (1:1:5)-etched surfaces.

#### 3.4.2 Crystal Quality Before and After Wet Etching

**Figure 4:**
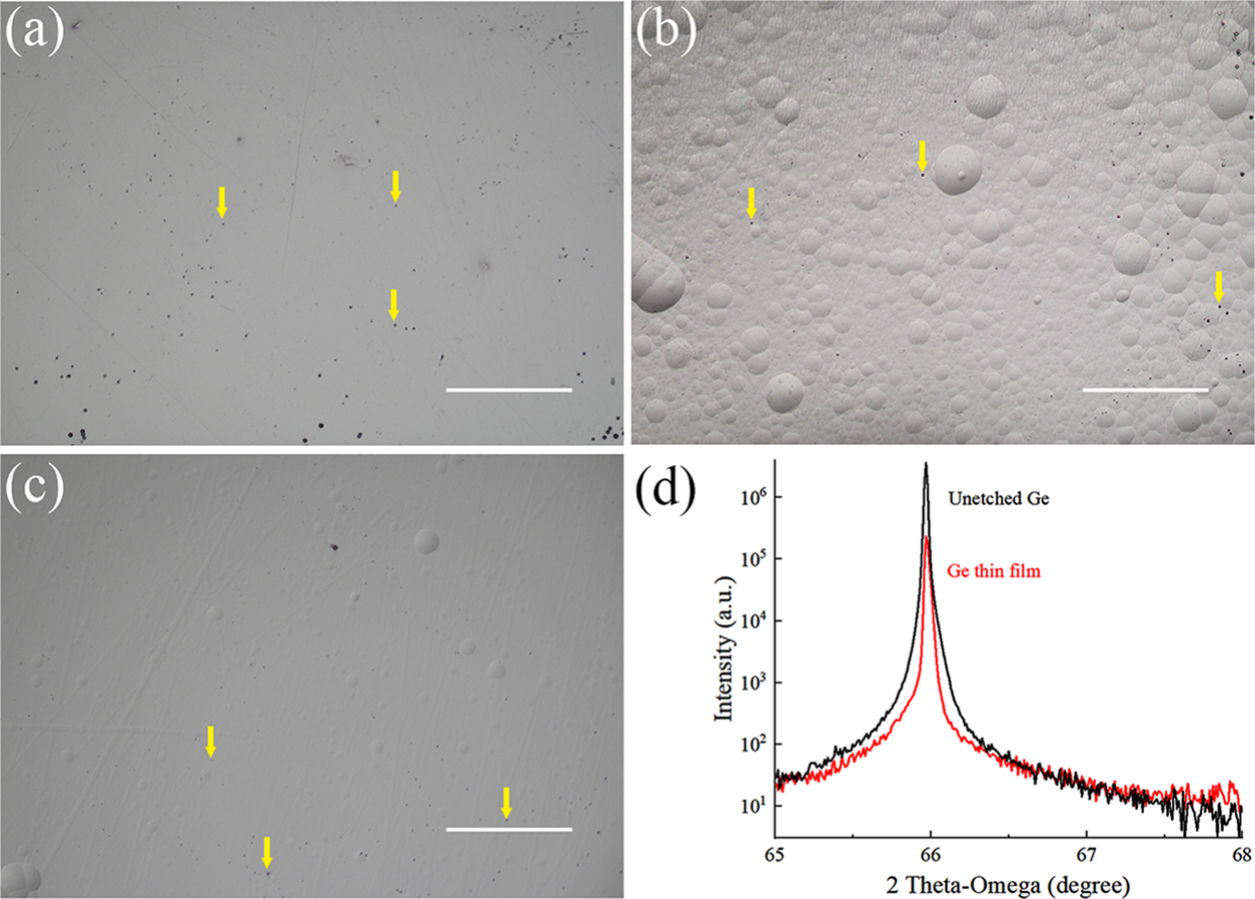
EPD results for (a) unetched Ge, (b) 57 h-nanostrip (1:1:10)-etched thin film, and (c) 53 h-HCl (1:1:5)-etched thin film. The yellow arrow points out the representation of the etching pit, and the scale bar is 500 μm. (d) HRXRD rocking curves of unetched bulk-Ge and the 53 h-HCl (1:1:5)-etched thin film.

The threading dislocation density before and after the etching processes was also checked with the EPD method discussed in 2.3, and the etch pits are shown in Figure 4a–c. The etching time was 90 s to get large enough pits for counting. The etching pit densities before and after the etching processes remained almost the same level of 6000–7000 cm^–2^.

The crystal quality was measured by HRXRD as shown in Figure 4d. Both the unetched and the 53 h-HCl (1:1:5)-etched thin film had a sharp Ge peak, indicating a good crystalline quality. The full width at half maximum (fwhm) of the Ge peak of HCl (1:1:5)-prepared thin film was 0.0269°, which increased a little bit from the 0.0192° of the unetched Ge. However, it was much better compared with epitaxial Ge on Si, which was reported to be 0.0736° in the literature.^15^ The Ge peak position stayed the same, and the peak shape was similar before and after the etching process, which also demonstrated that no strain or obvious lattice damage was introduced for the Ge thin film.

The key results and comparisons are summarized in Table 5 given below.

**Table 5:** Key Results and Comparison

	**virgin Ge**	**nanostrip** (1:1:10)	**HCl** (1:1:5)
Etch time (h)	0	57	53
Minimum thickness achieved (μm)		9.2	4.1
RMS and morphology	1.6 nm with small scratches	60 nm with big voids	10 nm with voids and deepened scratches
EPD	6000–7000 cm^–2^	6000–7000 cm^–2^	6000–7000 cm^–2^
HRXRD (fwhm)	0.0192°		0.0269°

### 3.5 Absorbance of the Ge Thin Film

**Figure 5:**
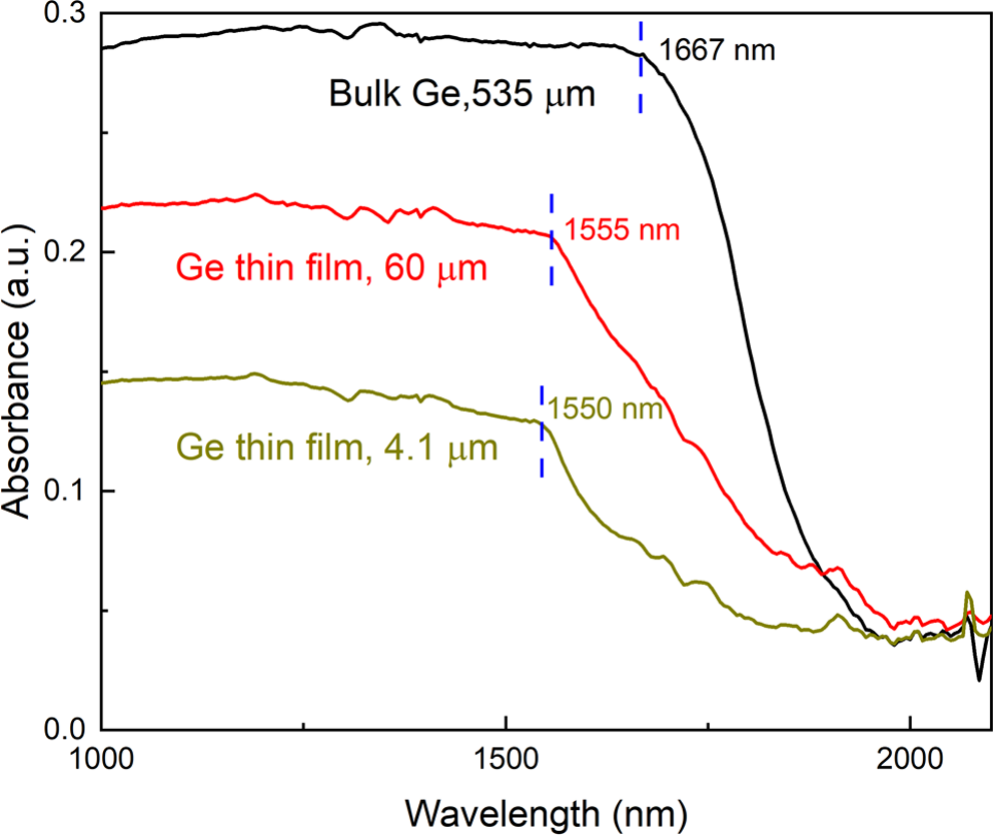
Absorbance of Ge with different thicknesses.

As mentioned before, one of the advantages of using a Ge thin film is the reduction in reabsorption as thickness decreases. This reduction is favorable for Ge’s light-emitting properties, as it allows more photons to escape from the material. To confirm this, we examined the absorbance of Ge with varying thicknesses, as shown in Figure 5. It can be seen that the absorbance decreased with the reduced thickness. Moreover, the absorption edge consistently shifts toward shorter wavelengths with smaller thicknesses. Initially, it was around 1667 nm for a bulk-Ge wafer, but it shifts to approximately 1550 nm when the Ge thickness is down to four microns. It is worth noting that Ge is an indirect-band gap material, and both the absorptions from the indirect band gap (0.66 eV, 1879 nm) and the direct band gap (0.8 eV, 1550 nm) contribute to the absorption spectrum. The transition at the indirect band requires the assistance of the phonon with the required momentum to bridge the offset between the conduction band minimum and valence band maximum.^35^ This mechanism results in a much lower probability of absorption compared to the direct band transition. Consequently, the absorption coefficient at the indirect band gap (1879 nm) is significantly smaller than that at the direct band gap (1550 nm) as mentioned in the ref (36).

However, it is essential to note that the contribution from the indirect-band gap absorption in bulk-Ge remains significant due to its substantial thickness, typically exceeding 500 μm. As the thickness decreases, the influence of the indirect-band gap absorption diminishes, resulting in a shift toward the wavelength associated with the direct band gap. When the thickness is reduced to less than 10 μm, the absorption from the indirect band gap is effectively suppressed, creating more opportunities for Ge to exhibit enhanced photoluminescence at 1550 nm.

### 3.6 Possible Mechanism for the Wet Etching of the Acidic H_2_O_2_ Solution

Ge wet etching with acidic H_2_O_2_ has been adopted for a very long time, and the related mechanisms both on the nanoscale and the atomic scale have been extensively studied for the research associated with Ge surface passivation,^37^ Ge surface cleaning,^38^ and Ge wet etching processes.^38^^32,39^ There are two different natural oxides, GeO and GeO_2_, on the surface of Ge.^40^ When H_2_O_2_ was applied, the oxide which is primarily water-soluble GeO_2_ will regrow. This oxide dissolves slowly in H_2_O and can be removed more rapidly by acids. With the continuous oxidation from H_2_O_2_ and oxide removal from acid, Ge could be thinned down. It should be noted that an anion like Cl^–^ may play a more important role in the etching process than proton concentration.^32,41^

Unlike prior studies which etched germanium with a short time and a limited depth, this work thinned Ge from the original thickness of 535 μm to a thickness of ≤10 μm. Therefore, it could be difficult to do the nanoscale mechanism investigation because the surface changed dramatically for such a long etching time (≥50 h). In this study, we only focused on the microscale morphology evolution of Ge wet etching for the long-time wet etching process.

#### 3.6.1 Roles of the Acid and H_2_O_2_

As shown in Tables 2–4, acids (HF, HCl, and H_2_SO_4_) were not able to thin Ge down without H_2_O_2_, which was consistent with the reports from the literature. The H_2_O_2_ solution (30%) alone was able to etch Ge with an etching rate of 2.5 μm/hs, but the surface roughness increased dramatically to 11 nm after the etching process (Figure S4). Adding acid into H_2_O_2_ increased the etching rate and improved the surface roughness. Thus, the etching process was considered to be a two-step process: (1) Ge was oxidized by H_2_O_2_ and (2) oxides were removed by H_2_O and acid.

**Figure 6:**
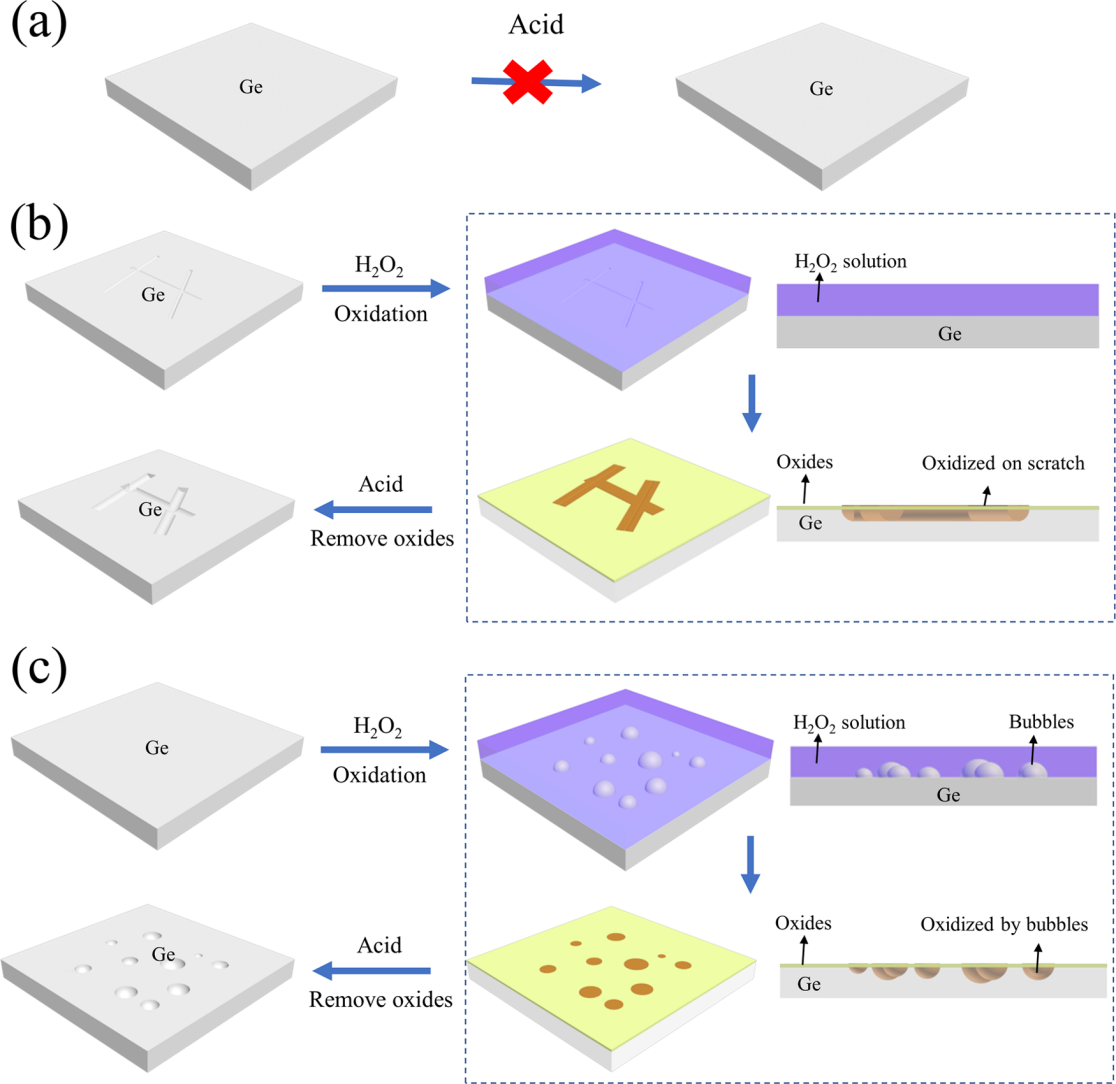
Illustration of Ge thinning mechanisms with acid and H_2_O_2_ solutions. (a) Ge cannot be thinned by a diluted acid alone, (b) scratch evolution with a diluted H_2_O_2_ and acid solution, and (c) bubble-induced void formation in a diluted H_2_O_2_ and acid solution.

Two types of defects could be seen after the wet etching: scratches and hemisphere voids. The scratches came from the original polishing traces (Figure 1b), which grew during the etching process as shown in Figure 6b. In addition to H_2_O_2_, a high-concentration H_2_SO_4_-like nanostrip-based solution (1:0:0) could also oxidize the surface, leaving scratches on the surface (Table 4). As for the hemisphere voids, these were more likely due to the O_2_ bubbles from the decomposition of H_2_O_2_. The O_2_ bubble is absorbed on the surface of Ge, gradually oxidizing Ge into oxides. After the oxides were removed by acid, hemisphere voids were generated, as shown in Figure 6c.

To confirm this, one drop of HCl (1:1:5) was put on the surface of Ge, and a video of the optical microscopy view was taken to check how the surface was changing during the etching process. One can see that some bubbles are absorbed on the surface and some move freely in the solution (Video 1). After 10 min of etching, the surface was cleaned with DI water, dried, and checked with the optical microscope (Figure S5). The surface roughness increased to 1.9 nm with the scratches deepened and some voids generated on the surface. With 30 min of etching, the surface was packed with voids and the surface roughness increased dramatically (Figure S5c and S5d). It should be noted that the real etching process (35 mL) was slightly different from the one-drop etching due to the larger volume.

#### 3.6.2 Role of the Volume Ratio

For a certain acidic H_2_O_2_ aqueous solution, say HCl-based solution, as the ratio changed gradually from 1:1:1 to 1:1:5 to 1:1:10 to 1:1:20, the etching rate decreased (Table 3), which could be attributed to the decreasing concentration for both H_2_O_2_ and acid. The surface etched with a high concentration solution (1:1:1) was rough, which could be due to more bubbles generated under a higher decomposition rate of H_2_O_2_ (Video 2). As the concentration of both acid and H_2_O_2_ decreased (ratio to 1:1:5), the surface roughness also decreased. However, as the concentration continued to drop to the ratios of 1:1:10 and 1:1:20, the surface roughness increased. A moderate concentration of the acid and H_2_O_2_ (such as HCl 1:1:5) might be preferred to realize a balance between the oxidation process (H_2_O_2_) and oxide removal process (acid).

#### 3.6.3 Role of Different Acids

When we compared the function of HCl and HF for the wet etching, with the same ratio of 1:1:1, the HF-based solution could reach a much higher etching rate. Therefore, the overall etching rate was controlled by the oxide removal rate for the HCl-based solution. A moderate oxide removing rate might favor the surface roughness, considering the long etching result for HCl (1:1:5).

HCl-based and nanostrip-based solutions exhibited a similar etching rate for ratios of 1:1:5, 1:1:10, and 1:1:20. The nanostrip-based solution showed lower surface roughness after 24 h of etching compared with the HCl-based solution under the same ratio. However, the thin film prepared by the nanostrip (1:1:10) had a much higher surface roughness (70 nm) than that of HCl (1:1:5), which might be due to the poor diffusion of H_2_SO_4_ in the solution. This was confirmed with the result that agitation could improve the surface roughness for the nanostrip (1:1:10)-prepared thin film (Figure 3a,3b,3d,3e). However, agitation did not improve the surface roughness etched by HCl (1:1:5) (Figure S3e and S3f), which demonstrated a good dispersion of ions in the HCl-based solution. This also indicated that the passivation of the Cl^–^ on the surface of Ge may be helpful for a uniform Ge etching process.

#### 3.6.4 Benefit of the Double-Sided Etching Setup

The double-sided etching could decrease the surface roughness after the etching process (Figure S3a, S3b, S3c, and S3d) because the bubbles were observed to attach to the Teflon stand surfaces, which decreased the nonuniformity from the bubbles on Ge surfaces (Figure S2).

## 4 Conclusions and Future Work

### 4.1 Conclusions

In this work, three different acidic–H_2_O_2_ solution types (HF-based, HCl-based, and nanostrip-based) with different volume ratios were studied and optimized in terms of postetching morphology, surface roughness, and etching rate. Both the nanostrip (1:1:10) and HCl (1:1:5) were able to wet-etch 535 μm-thick bulk-Ge substrates to 9.2 and 4.1 μm Ge films, respectively, which were the thinnest Ge films from bulk-Ge via a wet etching method to the best of our knowledge. The corresponding RMS surface roughness for the HCl-based solution-prepared thin film was 10 nm. The low threading dislocation density of 6000–7000 cm^–2^ was maintained in the process of wet etching without introducing extra defects. The good quality of the starting bulk-Ge was preserved after the etching process according to the HRXRD results. The etching mechanism and its implications were also thoroughly examined and discussed. This approach offers a cost-effective and convenient solution for precise Ge substrate thinning, making it suitable for various applications, including multijunction solar cells. Additionally, it facilitates the preparation of sub-10 μm-thick Ge thin films, thereby enabling further investigations into low-defect density Ge-based devices including photodetectors, LEDs, and lasers.

### 4.2 Future Work

Ge will be bonded on a substrate and undergo the wet etching thinning process. A polishing process may also be applied for a bonded Ge thin film on a handle substrate to obtain a lower surface roughness for future device (LEDs and lasers) fabrication.

## Supporting Information Available

Morphology of the 4 h-HF (1:1:10)-etched sample, photo of the Ge sample standing on a Teflon stand in the solution, optical images and 3D optical images of 24 h-HCl (1:1:5)-etched samples with single side etching, double-side etching, and double-side etching with agitation, and morphologies of 24 h-H_2_O_2_ solution (30%)-etched sample and one-drop HCl (1:1:5)-etched sample (PDF)
Bubbles absorbed on the surface and some moving freely in the solution (MP4)
Bubbles clearly seen in HCl (1:1:1) during the etching process (MP4)
