# Supplementary material for: Sub-10 μm-Thick Ge Thin Film Fabrication from Bulk-Ge Substrates via a Wet Etching Method
Source: ACS Omega. 2023 Dec 12;8(51):49201–10. doi: 10.1021/acsomega.3c07490 (PMC10753562; doi:10.1021/acsomega.3c07490)
Supplement: Supplementary file 1 — ao3c07490_si_001.pdf [file ao3c07490_si_001.pdf]

Supporting information

Sub-10-micron thick Ge thin film fabrication from bulk-Ge substrates via a wet etching method

Liming Wang[1], Ying Zhu[1,2], Rui-Tao Wen[2], and Guangrui (Maggie) Xia[1*]

[1]Department of Materials Engineering, The University of British Columbia, Vancouver BC, V6T 1Z4 Canada

[2]Department of Materials Science and Engineering, Southern University of Science and Technology, Shenzhen 518055, China

*Corresponding email: guangrui.xia@ubc.ca

Supplementary figures

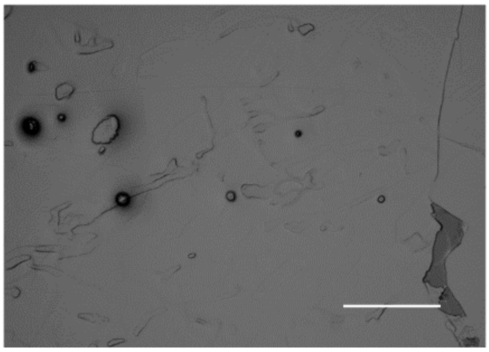

Figure S1. 4 h HF (1:1:10) etched sample, scale bar = 500 µm

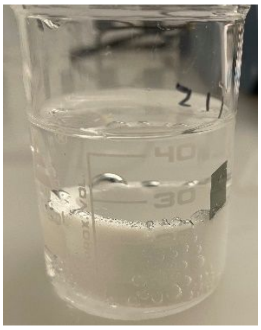

Figure S2. A photo of Ge sample standing on a Teflon stand in the solution

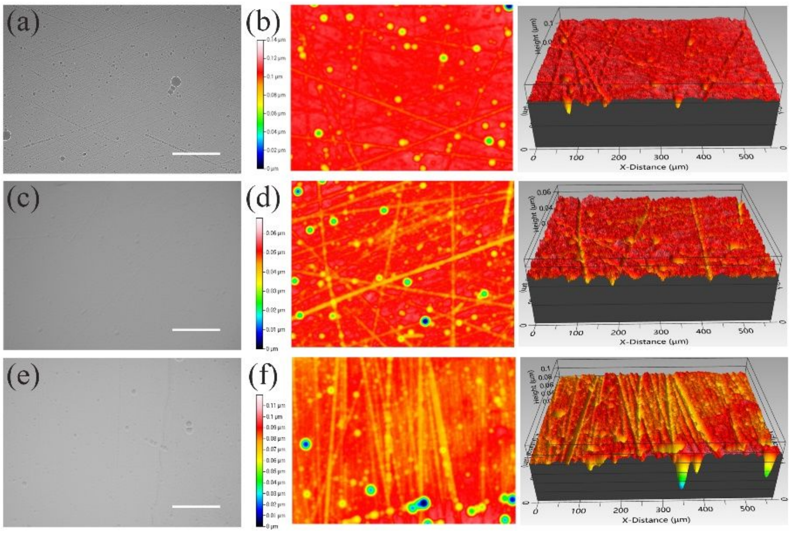

Figure S3. 24 h HCl (1:1:5) etched sample, (a) optical image of single side etched sample (b) optical interferometer image of single side etched sample, (c) optical image of double sides etched sample (d) optical interferometer image of double sides etched sample, (e) optical image of double sides etched sample, with agitation (f) optical interferometer image of double sides etched sample, with agitation

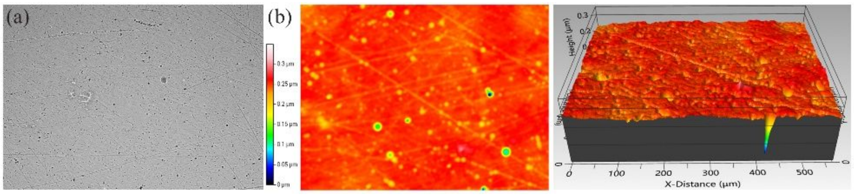

Figure S4. 24 h H_2O_2 solution (30 %) etched sample, (a) optical image (b) optical interferometer images, Sq = 11nm.

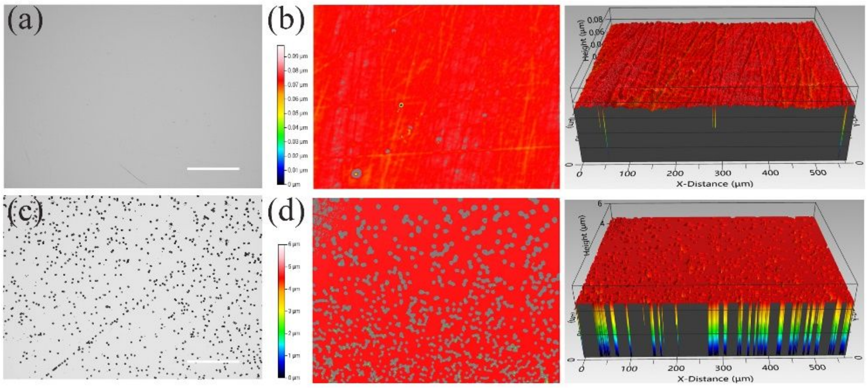

Figure S5. 1 drop HCl (1:1:5) etched sample, (a) optical image after 10 min etching (b) optical interferometer images after 10 min etching, Sq = 1.9 nm, (c) optical image after 30 min etching (d) optical interferometer images after 30 min etching
